# Comprehensive evaluation of immune dysregulation in secondary hemophagocytic lymphohistiocytosis

**DOI:** 10.1080/21505594.2024.2342276

**Published:** 2024-04-17

**Authors:** Yun Wang, Xu Yuan, Ting Wang, Wei Wei, Shiji Wu, Hongyan Hou

**Affiliations:** Department of Laboratory Medicine, Tongji Hospital, Tongji Medical College, Huazhong University of Science and Technology, Wuhan, China

**Keywords:** Secondary hemophagocytic lymphohistiocytosis (sHLH), laboratory parameters, immunological indicators, prognosis

## Abstract

Host immune dysfunction plays a crucial role in the onset, progression, and outcome of hemophagocytic lymphohistiocytosis (HLH). This study aimed to comprehensively evaluate the peripheral immune profiles in patients with newly diagnosed secondary hemophagocytic lymphohistiocytosis (sHLH), and explore their predictive value for patient prognosis. A total of 77 patients with sHLH were enrolled in this study, with 31 of them experiencing mortality. Flow cytometry was used to assess the percentages, absolute numbers, and phenotypes of lymphocyte subsets. Simultaneously, cytokine levels and routine laboratory indicators were also collected. In sHLH patients, lymphocyte subset absolute numbers were significantly impaired, accompanied by T cell hyperactivation, B cell hyperactivation, and increased plasmablast proliferation. Prognostic analysis revealed that lower CD8+ T cell percentages, elevated APTT, IL-6, IL-10 levels, and increased CD4+CD28null T cell proportions were associated with poor patient outcomes. The study demonstrates dysregulation in the counts and phenotypes of lymphocyte subsets in sHLH patients. Several key factors, including IL-6, IL-10, APTT, and various T cell percentages, have potential as prognostic markers and therapeutic targets in sHLH.

## Introduction

Hemophagocytic lymphohistiocytosis (HLH) is a rare and life-threatening syndrome characterized by a hyperinflammatory state associated with tissue lymphohistiocytic proliferation and hemophagocytosis [1–3]. It can be classified into primary HLH (pHLH) and secondary HLH (sHLH), with pHLH resulting from genetic defects and sHLH often triggered by infections, cancer, or autoimmune diseases. Infections associated with sHLH include Epstein-Barr virus, cytomegalovirus, herpes simplex virus, and varicella-zoster virus, while cancer-related sHLH can be linked to non-Hodgkin T or B lymphoma, and autoimmune diseases such as adult-onset Still’s disease (AOSD), systemic juvenile arthritis (sJIA), and systemic lupus erythematosus [4,5]. Given the highly mortality of the disease, prompt diagnosis and initiation of immunosuppressive therapy are crucial [6,7].

Fever, hepatosplenomegaly, cytopenias, hypofibrinogenemia, hypertriglyceridaemia, and elevated serum ferritin levels are characteristic features of HLH [8]. Although these findings collectively indicate HLH, many symptoms are non-specific, making early diagnosis challenging. Hyperactivation of macrophages and T lymphocytes is a prominent aspect of HLH, leading to T cell infiltrations in the liver, bone marrow, and brain, along with the systemic release of proinflammatory cytokines [9,10]. HLH is marked by overactivity in histiocytes and lymphocytes, resulting in a profound disruption of immune balance. Multiple pathways contribute to the development of this uncommon ailment. The disease is associated with rapid onset, rapid progression, and high mortality [11]. The underlying immune dysfunction plays a crucial role in the pathogenesis and outcome of HLH, particularly the impaired function of NK cells and cytotoxic T lymphocytes (CTLs) [8]. Primary HLH is associated with immune cell dysfunction resulting from genetic mutations and other hereditary factors. Nonetheless, the reasons behind the immune dysfunction in patients with sHLH remain unclear [12]. While studies have reported on lymphocyte subset analysis and immunophenotypic profiles in patients with pHLH [11,12] or a combination of pHLH and sHLH [13], there is a scarcity of data specific to sHLH patients [14,15]. The prevailing research predominantly emphasizes T cells [16,17] and NK cells [18], while giving comparatively less consideration to B cells. Regarding the prognosis of HLH, some studies have highlighted the significant prognostic value of sCD25 and ferritin [19–22]. Nevertheless, the utilization of a combination of immunological indicators to assess prognosis remained limited.

In our study, we thoroughly examined the immune profiles of newly diagnosed secondary hemophagocytic lymphohistiocytosis (sHLH) patients, comparing them to disease controls and healthy individuals. We also assessed the prognostic value of these profiles. Our focus was on analysing lymphocyte subsets using flow cytometry and collecting data on cytokines and routine laboratory indicators to understand immune dysregulation in sHLH and identify potential prognostic markers. This research could contribute to improved therapeutic strategies for sHLH patients.

## Materials and methods

### Patients

A total of 77 patients (39 males and 38 females) diagnosed with sHLH were included in this study from December 2021 to April 2023. All patients were aged >18 years, and the diagnosis of HLH was made using the criteria of the International HLH Society [8]. None of these patients had previous evidence for an underlying immunodeficiency, and none of them received previous immunosuppressive drugs. Moreover, 20 disease controls (DCs, 13 males and 7 females) and 36 healthy controls (HCs, 21 males and 15 females) were included in the study as age- and sex-matched comparison groups, determined by interview and physical examination. The DCs group included patients with infection, lymphoma at diagnosis, or autoimmune or inflammatory disease during disease flare-up, but without evidence of HLH. Peripheral blood samples were all taken at the time of diagnosis (within the first 24 hours) and before initiation of any specific treatment. The exclusion criteria include HIV and HCV positive. This study was approved by the ethical committee of Tongji Hospital, Tongji Medical College, Huazhong University of Science and Technology (TJ-IRB20230632).

### TBNK lymphocyte counting

The percentages and absolute numbers of CD4+ T cells, CD8+ T cells, B cells, NK cells and NKT cells were assessed using TruCOUNT tubes and BD Multitest 6-colour TBNK Reagent Kit (BD Biosciences) according to the manufacturer’s instructions. In brief, 50 mL of whole blood was labelled with six-colour TBNK Ab cocktail regent for 15 minutes at room temperature. Subsequently, 450 mL of FACS lysing solution was added, and the samples were analysed using a FACSCanto flow cytometer equipped with FACSCanto clinical software (BD Biosciences).

### Lymphocyte phenotypes, B cell, T helper (Th) cell and monocyte subsets analysis

The phenotypes of T cells and B cells were analysed by flow cytometry. Three panels were designed and the following monoclonal antibodies and reagents were added to 100 µl whole blood. The antibodies in panel 1 were anti-CD45-PerCP (BD Pharmingen, 2D1), anti-CD3-APC-H7 (BD Pharmingen, SK7), anti-CD4-V450 (BD Pharmingen, RPA-T4), anti-CD8-PE/Cy7 (BD Pharmingen, SK1), anti-CD28-PE (BD Pharmingen, L293), and anti-HLA-DR-APC (BD Pharmingen, L243). The antibodies in panel 2 were anti-CD45-PerCP (BD Pharmingen, 2D1), anti-CD3-APC-H7 (BD Pharmingen, SK7), anti-CD4-V500C (BD Pharmingen, SK3), anti-CD45RA-FITC (BD Pharmingen, L48), anti-CD8-PE/Cy7 (BD Pharmingen, SK1), anti-CCR7-PE (BD Pharmingen, 3D12), anti-CD25-APC (BD Pharmingen, 2A3), and anti-CD127-BV421 (BD Pharmingen, HIL-7 R-M21). The subsets of B cells analysed by panel 3 were detected using anti-CD38-FITC (BD Pharmingen, HB7), anti-CD19-PE/Cy7 (BD Pharmingen, SJ25C1), anti-CD27-PerCP (BD Pharmingen, 2D1) and CD45-V500C (BD Pharmingen, 2D1), and anti-IgD-APC (BD Pharmingen, IA6–2). For the subsets of monocytes, the following antibodies were used: anti-HLA-DR-FITC (BD Pharmingen, LN3), anti-CD16-PE (BD Pharmingen, B73.1), anti-CD14-APC (BD Pharmingen, MφP9), and anti-CD45-V500C (BD Pharmingen, 2D1). The subsets of Th cells were detected using anti-CD4-APC-Cy7 (BD Pharmingen, SK3), anti-CD25-APC (BD Pharmingen, 2A3), anti-CD127-BV421 (BD Pharmingen, HIL-7 R-M21), anti-CD45RA-BV510 (BD Pharmingen, HI100), anti-CXCR3-PE-Cy7 (BD Pharmingen, 1C6/CXCR3), anti-CCR6-PerCP-Cy5.5 (BD Pharmingen, 11A9). The subsets expressing perforin and granzyme B on NK, NK T, and CD8+ T cells were detected using anti-CD3-FITC (BD Pharmingen, SK7), anti-CD56-PE-Cy7 (BD Pharmingen, NCAM1), anti-CD8-BV605 (BD Pharmingen, HIT8a), anti-Perforin-PerCP-Cy5.5 (BD Pharmingen, B-D48), anti-Granzyme B-BV500 (BD Pharmingen, GB 11). Isotype controls with irrelevant specificities were included as negative controls. All of these cell suspensions were incubated for 20 min at room temperature. After lysing red blood cells with lysing solution, the pellets were washed, re-suspended in 300 ml PBS and then and analysed using a FACSCanto flow cytometer.

### Cytokine profile analysis

Serum samples were collected from all participants upon admission. The levels of IL-1β, IL-2 R, IL-8, IL-10, and TNF-α were determined using an automated solid-phase two-site chemiluminescent immunometric assay on the IMMULITE 1000 Analyzer (Siemens). The level of IL-6 was measured using the electrochemiluminescence method with Roche Diagnostics. These assays were performed according to the manufacturer’s instructions to ensure accurate and reliable measurements of cytokine levels in the serum samples.

### Statistical analysis

Continuous variables were presented as either mean ± standard deviation (SD) or median (interquartile range (IQR)), depending on the data distribution. The Mann-Whitney U test or one-way ANOVA test was used to compare the variables between groups, as appropriate. Categorical variables were compared using the Chi-square test or Fisher’s exact test, depending on the sample size and expected cell frequencies. These tests were employed to analyse the associations between categorical variables in the study. Kaplan-Meier curves were used for survival analysis and compared by using log-rank test. The prognostic factors of 28-day overall survival (OS) were analysed by Cox regression model. Unsupervised hierarchical cluster analysis was conducted to identify clusters of patients exhibiting similar immune patterns. This analysis was performed using the R package “pheatmap,” and the results were represented as a dendrogram. Statistical analyses were performed using GraphPad Prism version 9.5 (San Diego, CA, USA) and SPSS version 22.0 SPSS, Chicago, IL, USA). Statistical significance was determined as *p* < 0.05.

## Results

### Participant characteristics

This study recruited 77 sHLH patients with the median age of 59 (25th-75th, 44–66) years. Meanwhile, 36 healthy controls, including 21 males and 15 females, were also included, with a median age of 58 years (25th −75th, 52–59). Additionally, 20 patients with infections or tumours were recruited as DCs group. The main clinical and biological characteristics of the participants are summarized in Table 1. Clinical manifestations such as fever and splenomegaly were observed in the majority of patients with sHLH. Regarding the underlying diseases, the majority of sHLH cases were associated with viral infections, with notable pathogens including Epstein-Barr virus (EBV), cytomegalovirus (CMV), and severe fever with thrombocytopenia syndrome virus (SFTSV). Patients with sHLH had significantly lower white blood cell (WBC) counts, platelet counts, and lymphocyte levels compared to the DC groups. Blood chemistry analysis revealed that individuals with sHLH had elevated levels of liver enzymes, bilirubin, gamma-glutamyl transferase (GGT), lactate dehydrogenase (LDH), triglycerides, and ferritin (*p* < 0.05). Additionally, sHLH patients exhibited reduced fibrinogen (FIB) levels and increased thrombin time (TT) and D-dimer levels (*p* < 0.001). Cytokine analysis showed that sHLH patients had higher levels of IL-2 R, IL-6, IL-10, and TNF-α (*p* < 0.001).

**Table 1.** Clinical and biological characteristics.

Parameters	HLH (*n* = 77)	DC (*n* = 20)	*p* value
Age, median (IQR), year	59 (46–66)	56 (51–60)	0.649
Sex			
Male, n (%)	39 (50.6%)	13 (65.0%)	0.252
Female, n (%)	38 (49.4%)	7 (35.0%)	
Secondary disease, n (%)			
Lymphoma	9 (11.7%)	1 (5.0%)	0.643
Bacterial, parasite, fungus	8 (10.4%)	5 (25.0%)	0.180
AID	1 (1.3%)	6 (30.0%)	<0.001
Virus (EBV, CMV, SFTSV, HBV)	53 (68.8%)	8 (40.0%)	0.017
Idiopathic	6 (7.8%)	0 (0%)	0.443
**Blood routine indicators**			
WBC (x10^9^/L)	3.99 (1.73–8.03)	7.55 (4.61–9.86)	0.010
Lymphocytes (x10^9^/L)	0.62 (0.40–1.18)	1.52 (1.31–2.19)	<0.001
RBC (x10^12^/L)	3.55 (2.93–4.17)	3.94 (3.59–4.28)	0.073
Hemoglobin (g/L)	106.00 (83.00–123.00)	117.50 (106.50–131.75)	0.027
Platelet (x10^9^/L)	49.00 (30.00–84.00)	205.50 (118.75–274.00)	<0.001
**Blood biochemistry indicators**			
ALT (U/L)	64.00 (34.00–144.00)	26.00 (17.75–74.00)	0.010
AST (U/L)	129.00 (59.00–303.00)	25.00 (19.00–62.00)	<0.001
Total protein (g/L)	57.40 (48.75–65.25)	65.95 (62.03–70.83)	<0.001
Albumin (g/L)	30.80 (27.45–34.10)	34.35 (29.85–37.43)	0.019
TBIL (μmol/L)	13.50 (8.75–30.30)	9.40 (5.45–16.60)	0.016
DBIL (μmol/L)	6.80 (4.70–21.00)	4.70 (2.78–7.25)	0.007
IBIL (μmol/L)	5.60 (3.35–8.20)	4.35 (2.98–7.33)	0.283
Amylopsin (U/L)	112.00 (81.50–268.50)	98.00 (76.25–156.50)	0.220
GGT (U/L)	102.00 (44.50–212.50)	46.50 (37.25–89.25)	0.034
LDH (U/L)	744.00 (469.00–1436.00)	206.00 (165.75–275.50)	<0.001
Triglyceride (mmol/L)	3.31 (1.58–3.89)	1.23 (1.05–1.45)	0.005
Ferritin (μg/L)	9281.00 (2388.55–23358.50)	577.80 (327.25–767.15)	<0.001
**Coagulation markers**			
PT (s)	14.20 (13.10–15.90)	13.85 (13.40–15.18)	0.913
FIB (g/L)	2.26 (1.58–3.02)	4.62 (3.08–5.79)	<0.001
APTT (s)	53.10 (38.10–66.50)	43.70 (39.83–48.18)	0.119
TT (s)	22.20 (18.70–35.90)	17.10 (16.23–18.18)	<0.001
D-dimer (μg/mL)	4.89 (2.28–11.30)	1.58 (1.09–2.36)	<0.001
**Cytokine indicators**			
IL-1β (pg/mL)	5.75 (5.00–11.25)	5.00 (5.00–6.90)	0.302
IL-2 R (U/mL)	2156.50 (1457.25–7355.25)	845.50 (602.00–1328.00)	<0.001
IL-6 (pg/mL)	55.80 (14.90–99.46)	14.80 (10.89–22.84)	0.005
IL-8 (pg/mL)	34.25 (22.23–80.40)	19.60 (12.53–57.18)	0.114
IL-10 (pg/mL)	63.70 (22.53–130.00)	5.00 (5.00–7.18)	<0.001
TNF-α (pg/mL)	28.40 (18.60–46.40)	13.70 (11.93–16.93)	<0.001

Data are presented as number (%), or median (25th − 75th percentile). HLH, hemophagocytic lymphohistiocytosis; DC, disease control; AID, autoimmune disease; EBV, Epstein-Barr virus; CMV, cytomegalovirus; SFTSV, severe fever with thrombocytopenia syndrome virus; HBV, hepatitis B virus; WBC, white blood cells; RBC, red blood cells; ALT, alanine aminotransferase; AST, aspartate aminotransferase; TBIL, total bilirubin; DBIL, direct bilirubin; IBIL, indirect bilirubin; GGT, gamma-glutamyl transferase; LDH, lactate dehydrogenase; PT, prothrombin time; FIB, fibrinogen; APTT, activated partial thromboplastin time; TT, thrombin time; IL, interleukin; TNF, tumour necrosis factor; Viral infections: 53 of 77 HLH patients (including 14 with EBV, 5 with CMV, 1 with HBV and 33 with SFTSV) and 8 of 20 DCs.

### The comparison of immune characteristics between patients with sHLH and control groups

**Figure 1.**
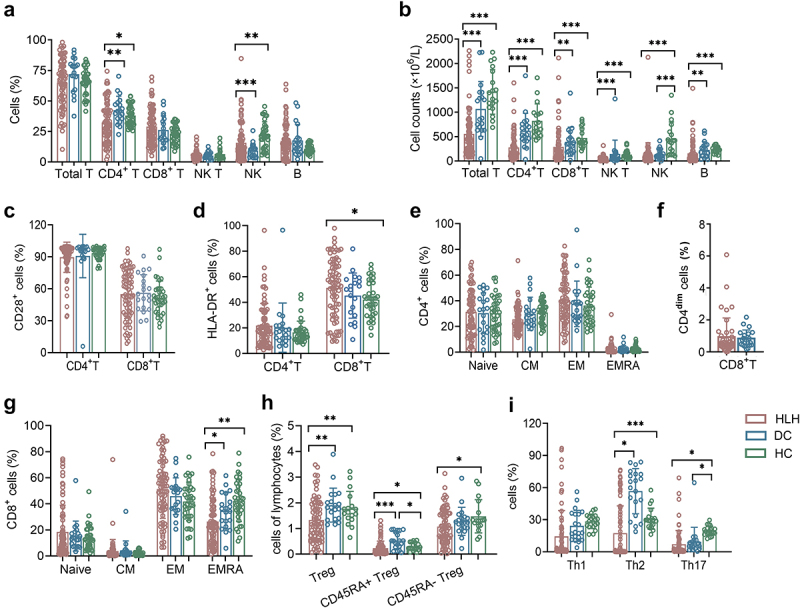
The lymphocyte subsets and immunophenotype characteristics. Circulating lymphocytes in patients newly diagnosed with secondary hemophagocytic lymphohistiocytosis (sHLH), disease controls (DCs) and healthy controls (HCs) were analysed using flow cytometer. (a,b) the percentages and absolute numbers of T cells, B cells, NK cells and NK T cells in different groups were expressed as mean with standard deviation (SD). (c,d) the percentages of HLA-DR and CD28 positive cells in CD4+ and CD8+ T cells from sHLH patients, DCs and HCs were expressed as mean with SD. (e–g) the percentages of CD4+ T and CD8+ T cell subtypes in different groups were expressed as mean with SD. (h,i) the percentages of Treg cells and Th cells subtypes in different groups were expressed as mean with SD. HLH, hemophagocytic lymphohistiocytosis; DCs, disease controls; HCs, healthy controls; Blue circle points represent DCs, green circle points represent HCs, and red circle points represent HLH patients. *p < 0.05, **p < 0.01, ***p < 0.001, ****p < 0.0001.

In sHLH patients, significant alterations were observed in the percentages of immune cell subsets when compared to both HCs and DCs. Specifically, the percentages of CD4+ T cells were found to be decreased compared to both HCs and DCs. Similarly, the percentages of NK cells were higher when compared to DCs, but lower when compared to HCs. These findings indicate a dysregulation of immune cell populations in sHLH. Furthermore, analysis of lymphocyte subset absolute numbers revealed significant impairments in sHLH patients compared to HCs and DCs (Figure 1(a–b)). In terms of specific immune cell phenotypes, sHLH patients exhibited distinctive characteristics. The expression of HLA-DR on CD8+ T cells was found to be increased in sHLH patients compared to HCs, but it did not differ significantly from DCs. There was no notable difference observed between HCs and DCs (Figure 1(d)). To identify the naïve/memory phenotypes of T cells, the expression of CCR7 and CD45RA was analysed. The results revealed a reduced percentage of terminally differentiated effector CD8+ T cells (TEMRA subtype, CCR7-CD45RA+) compare to both DCs and HCs. No significant differences were observed in the CD4+ T cell subtypes. Similary, the percentage of CD4dimCD8+ T cells showed no significant difference when compared to DCs (Figure 1(e–g)). The percentages of regulatory T (Treg) cells and CD45RA+ Tregs in sHLH patients were significantly decreased compared to both DCs and HCs. Additionally, the percentage of CD45RA- Treg cells was also decreased compared to HCs (Figure 1(h)). Furthermore, sHLH patients exhibited lower proportions of Th2 cells and Th17 cells when compared to HCs. When compared to DCs, the proportions of Th2 cells decreased significantly (Figure 1(i)).

**Figure 2.**
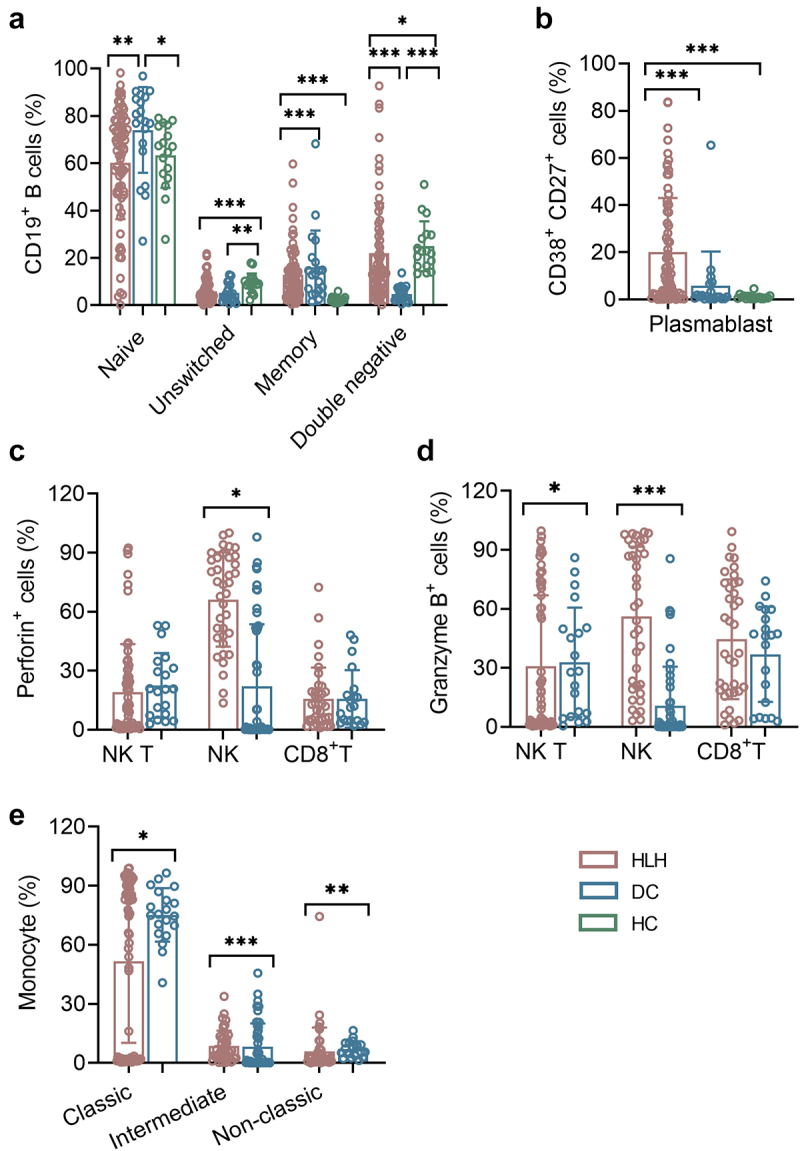
The subsets of B cells, NK, NK T, CD8+ T cells and monocytes cells. (a,b) the percentages of B cell Naïve (IgD+ CD27−), unswitched (IgD+ CD27+), memory (IgD− CD27+), and plasma cells (CD27+ CD38high) from HLH patients, DCs and HCs were expressed as mean with SD. (c,d) the expression of perforin and granzyme B on NK, NK T and CD8+ T cells form HLH patients and DCs were expressed as mean with SD. (e) The percentages of monocyte subsets in HLH and DCs groups were expressed as mean with SD. HLH, hemophagocytic lymphohistiocytosis; DCs, disease controls; HCs, healthy controls; Blue circle points represent HCs, brown circle points represent DCs, and red circle points represent HLH patients. *p < 0.05, **p < 0.01, ***p < 0.001, ****p < 0.0001.

Patients with sHLH displayed substantial changes in B cell subpopulations (Figure 2(a–b)). The unswitched B cells (CD27+IgD+) were significantly reduced in sHLH patients compared to healthy controls (HCs), while memory B cells (CD27+IgD-), double negative B cells, and plasma B cells (CD27+CD38+) showed marked increases (10.500 (2.090, 32.280) vs 0.470 (0.290, 0.990)). In comparison to disease controls (DCs), naive B cells (CD27-IgD+) and memory B cells decreased (66.900 (48.020, 77.850) vs 78.600 (68.400, 87.100); 10.100 (4.600, 16.240) vs 13.200 (6.300, 18.200)), while plasma B cells and double negative B cells exhibited significant increases.

The numbers of NK cells displayed a decreasing trend from HCs to DCs and sHLH groups. The expression of perforin on NK cells was significantly increased in sHLH patients. Additionally, both NKT cells and NK cells exhibited high expression levels of granzyme B (Figure 2(c–d)). The analysis of monocyte subsets revealed that the intermediate (CD14++CD16+) and non-classic (CD14+ CD16+) subsets were decreased in sHLH patients, with the classic (CD14++ CD16-) subset was increased when compared to DCs (Figure 2(e)). The numerical values for these findings are provided in Supplementary Table S1. A hierarchically clustered heatmap was developed for sample visualization, indicating the impairment of adaptive immune response (Supplementary Figure S1).

### Prognostic factors of sHLH

**Figure 3.**
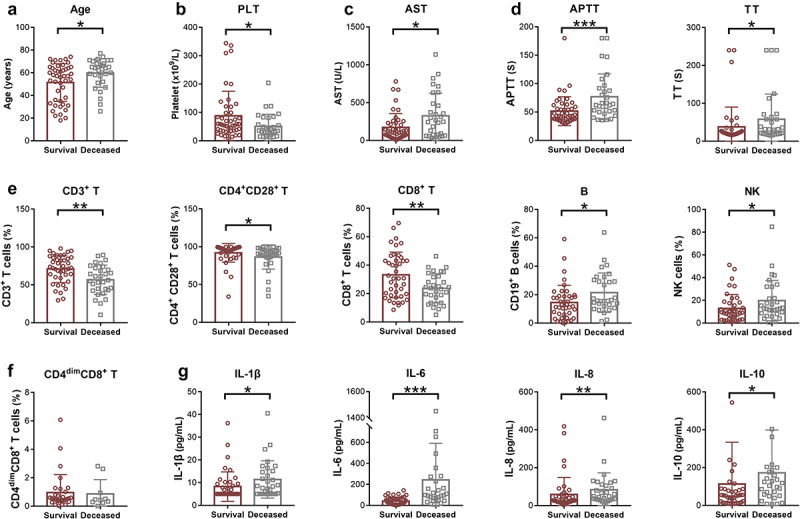
The differences of laboratory and immune indicators between deceased and survival HLH patients. (a) The age and blood indicators with significant differences between deceased patients and survivors were shown. (b) The immune indicators showed variations between the survival and deceased groups. (c) Cytokine indicators demonstrated differences between the survival and deceased group. HLH, hemophagocytic lymphohistiocytosis. PLT, platelet; AST, aspartate aminotransferase; APTT, activated partial thromboplastin time; TT, thrombin time; IL, interleukin. *p < 0.05, **p < 0.01, ***p < 0.001, ****p < 0.0001.

To investigate the potential prognostic markers of sHLH, laboratory and immune indicators were compared between deceased and survival patients in the following 28 days from disease onset. Table 2 presents the clinical and biological characteristics of the survival and deceased groups in the study. The median age in the survival group was 57 years (25th −75th, 35–64), while in the deceased group, it was 64 years (25th −75th, 55–68), showing a significant difference (*p* = 0.024). In terms of clinical manifestations, both groups had a high prevalence of fever. Among the blood routine indicators, platelet count was significantly lower in the deceased group (*p* = 0.025). Regarding blood biochemistry indicators, aspartate aminotransferase (AST) levels were significantly higher in the deceased group compared to the survival group (*p* = 0.021). However, no significant differences were observed in ALT, LDH, and ferritin levels between the two groups. In terms of coagulation markers, the APTT and TT were significantly prolonged in the deceased group compared to the survival group (Figure 3(b–d)). As for immune indicators, the percentages of CD3+ T cells, CD4+ CD28+ T cells and CD8+ T cells were all deceased in the deceased group compared to the survival group. On the other hand, the percentages of B cells and NK cells were increased in the deceased group (Figure 3(e)). The percentage of CD4dim CD8+ T cells showed no significant difference between the two groups (Figure 3(f)). The levels of IL-1β, IL-6, IL-8, and IL-10 were significantly higher in the deceased group (Figure 3 (g)).

**Table 2.** Clinical and biological characteristics between survival and deceased groups.

Parameters	Survival (*n* = 46)	Deceased (*n* = 31)	*p* value
Age, median (IQR), year	57 (35–64)	64 (55–68)	0.024
Sex			
Male, n (%)	23 (50.0%)	16 (51.6%)	0.89
Female, n (%)	23 (50.0%)	15 (48.4%)	
Clinical, n (%)			
Fever	41 (89.1%)	31 (100.0%)	0.154
Splenomegaly	24 (52.2%)	7 (22.6%)	0.009
**Blood routine indicators**			
WBC (x10^9^/L)	4.16 (2.01–7.78)	2.90 (1.57–7.30)	0.406
Lymphocytes (x10^9^/L)	0.61 (0.40–1.10)	0.62 (0.42–1.29)	0.787
RBC (x10^12^/L)	3.45 (2.96–3.91)	3.81 (2.96–4.51)	0.201
Hemoglobin (g/L)	101.00 (82.50–116.00)	115.00 (87.50–131.50)	0.140
Platelet (x10^9^/L)	57.50 (36.25–98.75)	36.00 (23.50–69.00)	0.025
**Blood biochemistry indicators**			
ALT (U/L)	66.00 (34.00–149.25)	63.00 (42.50–117.00)	0.876
AST (U/L)	107.50 (58.00–226.25)	247.00 (69.00–472.50)	0.021
LDH (U/L)	643.50 (421.25–1179.00)	875.00 (630.00–1467.50)	0.126
Ferritin (μg/L)	8332.40 (2255.88–21688.25)	13601.50 (2610.93–24615.50)	0.377
**Coagulation markers**			
PT (s)	13.85 (13.03–15.90)	14.40 (13.45–15.80)	0.38
FIB (g/L)	2.24 (1.53–2.88)	2.30 (1.68–3.10)	0.394
APTT (s)	43.15 (36.78–58.20)	62.20 (50.65–89.60)	0.001
TT (s)	21.80 (18.18–26.20)	30.80 (19.40–60.75)	0.049
D-dimer (μg/mL)	3.77 (2.30–11.35)	5.51 (2.46–10.14)	0.598
**Cytokine indicators**			
IL-1β (pg/mL)	5.00 (5.00–9.20)	8.40 (5.00–14.98)	0.014
IL-2 R (U/mL)	2420.50 (1461.00–7497.00)	2070.00 (1479.50–5444.25)	0.743
IL-6 (pg/mL)	26.62 (11.50–64.38)	97.16(53.28–262.03)	<0.001
IL-8 (pg/mL)	26.75 (18.58–48.70)	54.25 (33.75–118.50)	0.001
IL-10 (pg/mL)	50.20 (14.65–84.83)	111.00 (52.88–184.00)	0.011
TNF-α (pg/mL)	25.60 (16.48–38.10)	34.85 (22.25–67.98)	0.051

Data are presented as number (%), or median (25th − 75th percentile). WBC, white blood cells; RBC, red blood cells; ALT, alanine aminotransferase; AST, aspartate aminotransferase; LDH, lactate dehydrogenase; PT, prothrombin time; FIB, fibrinogen; APTT, activated partial thromboplastin time; TT, thrombin time; IL, interleukin; TNF, tumour necrosis factor.

**Figure 4.**
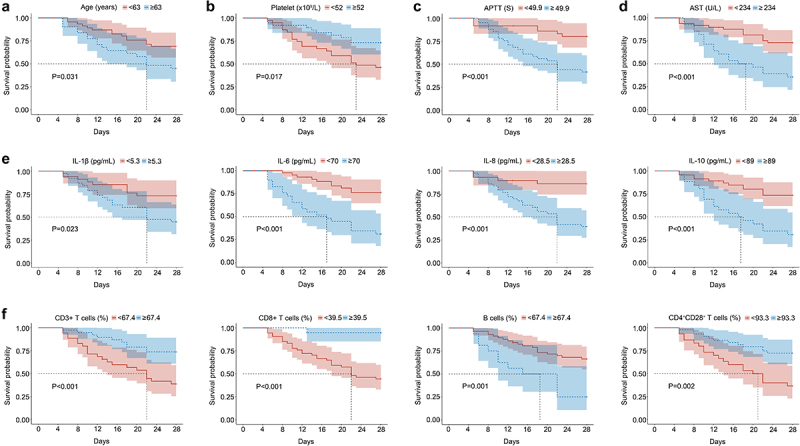
Kaplan-Meier curves. (a) Following age and blood indicators were compared. (b) Following the cytokine indicators were compared. (c) Following the immune indicators were compared. APTT, activated partial thromboplastin time; AST, aspartate aminotransferase; IL, interleukin.

Kaplan-Meier survival analysis demonstrated that advanced age (*p* = 0.031), elevated APTT levels (*p* < 0.001), higher levels of IL-6 (*p* < 0.001), IL-10 (*p* < 0.001), as well as reduced percentages of CD3+ T cells (*p* < 0.001), CD8+ T cells (*p <* 0.001), and CD4+CD28+ T cells (*p* < 0.001) were associated with poor prognosis (Figure 4). Furthermore, the univariate Cox regression analysis revealed that several factors were significantly associated with poor overall survival (OS) in sHLH patients. These factors included advanced age (*p* = 0.025), high levels of AST (*p* = 0.010), IL-1β (*p* = 0.041), IL-6 (*p* < 0.001), IL-8 (*p* = 0.021), IL-10 (*p* = 0.150), APTT (*p* < 0.001), and the percentage of B cells (*p* = 0.013) and NK cells (*p* = 0.057). Conversely, low levels of platelet (*p* = 0.062), the percentage of CD3+ T cells (*p* = 0.002), CD8+ T cells (*p* = 0.015) and CD4+CD28+ T cells (*p* = 0.069) were also significantly correlated with poor OS. Subsequently, the multivariate Cox regression analysis identified IL-6 (*p* < 0.001), IL-10 (*p* = 0.115), APTT (*p* = 0.084), and the percentage of CD8+ T cells (*p* = 0.042) and CD4+CD28+ T cells (*p* = 0.078) as independent risk factors for OS in sHLH patients (Table 3).

**Table 3.** Univariate and multivariate cox regression analysis of overall survival.

	Univariate cox regression			Multivariate cox regression		
Variables	HR	95% CI of HR	*p* value	HR	95% CI of HR	*p* value
Age (years)	1.031	1.004–1.059	0.025			
Platelet (x10^9^/L)	0.992	0.984–1.000	0.062			
AST (U/L)	1.001	1.000–1.003	0.010			
IL-1β (pg/mL)	1.042	1.002–1.085	0.041			
IL-6 (pg/mL)	1.003	1.002–1.004	<0.001	1.003	1.001–1.004	<0.001
IL-8 (pg/mL)	1.001	1.000–1.001	0.021			
IL-10 (pg/mL)	1.001	1.000–1.002	0.150	1.001	1.000–1.003	0.115
APTT (s)	1.015	1.007–1.022	<0.001	1.008	0.999–1.017	0.084
TT (s)	1.005	1.000–1.009	0.062			
CD3^+^ T cells %	0.973	0.957–0.990	0.002			
B cells %	1.028	1.006–1.052	0.013			
CD8^+^ T cells %	0.966	0.939–0.993	0.015	0.964	0.931–0.999	0.042
NK cells %	1.018	0.999–1.037	0.057			
CD4^+^ CD28^+^ T cells %	0.982	0.962–1.001	0.069	0.978	0.955–1.002	0.078

AST, aspartate aminotransferase; APTT, activated partial thromboplastin time; TT, thrombin time; IL, interleukin.

## Discussion

Hyperactivation of the immune system, hypercytokinemia, and severe systemic inflammation typically defIne the occurrence of HLH. A comprehensive and systematic evaluation of the host immune status in sHLH is essential for better understanding its pathogenesis and early identification of disease occurrence and risk stratification.

Our study demonstrated the dysregulation of lymphocyte subsets in sHLH patients, accompanied with increased percentage of CD8+ T cells but with decreased percentages of CD4+ T cells and NK cells, which were consistent with previous research findings [23,24]. In the early stage of adult secondary HLH patients, T cells that exert cellular immunity function are mainly CD8+T cells that are activated and proliferated under antigen stimulation [25], which significantly increases the percentages of T cells and CD8+ T cells. Meanwhile, the absolute numbers of whole lymphocyte subsets were significantly impaired in sHLH group. The reasons may be various, including macrophage activation, senescence and apoptosis caused by T cell activation, and secondary diseases, such as the immunosuppressive state of tumour, sepsis and other diseases [26–29]. The expression of HLA-DR on CD8+ T cells was found to be increased, indicating an activated immune response in sHLH patients. The research conducted by Nguyen et al. [17] has demonstrated that quantifying T-cell activation accurately can be achieved by directly measuring T-cell activation using flow cytometry, specifically through the identification of HLA-DR+CD38hi T cells, a subset first reported by the Chaturvedi et al. [30] for distinguishing between HLH and sepsis. Furthermore, this method exhibits a strong correlation with sIL-2 R levels across a spectrum of hyperinflammatory and immune dysregulation disorders. Therefore, the increased presence of HLA-DR+CD8+ T cells, indicating the overactivation of cytotoxic T lymphocytes, is considered one of the key factors contributing to immune dysregulation in sHLH. In addition, the frequency of CD4dimCD8+ T cells which is a subset of HLA-DR+CD38hi T cells significantly correlated with a clinical severity score in patients with sHLH [31]. Furthermore, the frequency of CD4dimCD8+ T cells in sHLH was indeed slightly higher than in DCs, but this difference did not reach statistical significance. This discrepancy may be attributed to differences in the study population. Our study exclusively focused on adults (all participants were over 40 years old), whereas many other studies primarily included children or adolescents [16,17,31]. It’s worth noting that age itself is positively associated with the activation of T cells [32].

The dysregulation of B cell subpopulations in sHLH patients is a significant observation, which indicate a disturbance in the maturation and differentiation processes of B cells. In sHLH patients, the percentages of naïve B cells and unswitched B cells were found to be significantly decreased. Conversely, the percentages of memory B cells, plasma B cells and double negative B cells were markedly increased in HLH patients. Previous studies have indicated that the absolute count of B cells in HLH patients is decreased, which was consistent with our findings. However, in contrast to our results, the number of plasma B cells is reduced [33]. The discrepancy may arise from the fact that a majority of our HLH patients were infected with SFTSV, resulting in an immunophenotype marked by heightened levels of plasma cells [34]. Unswitched B cells are considered to be naïve or early memory B cells. Memory B cells play a crucial role in generating specific immune responses upon re-exposure to antigens, whereas plasma cells are responsible for antibody production. Double negative B cells refer to antigen-experienced mature B cells that exhibit heterogeneity in terms of double-negative (DN) subsets, functional characteristics, and developmental properties [35]. Notably, they are associated with autoimmune conditions and a heightened inflammatory status [36].

Decreased or absent NK activity is a hallmark and one of the diagnostic criterions of HLH [8]. This criterion relates to NK function [37], but the percentage of NK is also affected. Interestingly, we also observed increased expressions of perforin and granzyme B on NK cells in sHLH patients compared to DCs. Additionally, the expressions of granzyme B on NKT cells were also elevated. Perforin plays an crucial role in cytotoxic activity of NK and cytotoxic CD8+ T cells [38]. NK cells exhibit spontaneous cytotoxicity towards target cells, which relies on the release of perforin and granzyme B [39]. Previous studies have reported that perforin expression, degranulation, and cytotoxic function of NK cells are generally normal in sHLH patients. However, some studies have reported that increased detection of granzyme B in both CTL and NK are a signature of HLH-associated immune activation [40]. The observed expression of perforin and granzyme B might exhibit discrepancies with the cytotoxicity as determined by K562 cell assays. Therefore the utility and explanation of perforin and granzyme B in sHLH require further investigation. The above cellular immune dysregulation is reversible through the control of T cell activation and excessive inflammation [33]. In addition to steroids, targeted therapies, such as JAK-STAT inhibitors [41] and the IFN-γ blocking antibody emapalumab [42], could partially reverse the effects of IFN-γ on B cell development, thereby potentially improving humoral immune responses.

Multivariate Cox analysis demonstrated significant correlations between the levels of IL-6, IL-10, APTT, the percentage of CD8+ T cells, CD4+CD28+ T cells, and the 28-day overall survival (OS) in patients with sHLH. Elevated levels of both IL-6 and IL-10 signifying high inflammation concurrent with an immunosuppressive status [43], prolonged APTT (dysregulation of coagulation), depletion of host immune cells (primarily CD8+ T cells), and the presence of CD4+CD28null senescent T cells were more pronounced in deceased patients compared to survivors. CD4+CD28null senescent T cells are profoundly proinflammatory, possessing the functional characteristics of cytotoxic lymphocytes, and their population can expand from less than 1% to over 50% of the total CD4+ T cell population [44]. Additionally, they serve as prognostic factors in conditions like autoimmune disease [45] and cardiovascular diseases [46]. Previous studies on the prognosis of HLH have indicated that sCD25 [21,22] or ferritin [20], or the combined use of sCD25 and ferritin [19], holds significant prognostic value for HLH. However, our research reveals that the levels of sCD25 and ferritin did not exhibit significant differences between the deceased and survival patient groups. The variations in prognostic markers across studies can stem from differences in study populations, disease heterogeneity, detection platforms, marker selection, sample size, study design, validation methods and temporal changes.

While our study provides valuable insights into immune dysregulation in sHLH, it has limitations. Notably, we did not measure the interferon-gamma/CXCL9 axis, a central pathway in HLH pathogenesis [47]. Additionally, the study design prevents establishing causal relationships between immune profiles and prognosis. The heterogeneity in underlying conditions, such as malignancy-associated HLH and EBV-associated HLH, which have poor prognosis [48] introduces complexity, and caution is required when interpreting results. Further investigations are needed to elucidate the underlying mechanisms and gain a better understanding of the functional implications of altered immune cell subsets in the context of sHLH.

## Conclusion

In conclusion, our study provided a comprehensive analysis of the immune landscape in sHLH. Our findings demonstrated dysregulation within the lymphocyte subsets in the sHLH group, with notable impairments observed in the absolute numbers of these subsets. Importantly, we identified several prognostic factors, including IL-6, IL-10, APTT, and the proportions of CD8+ T cells and CD4+CD28+ T cells, that hold significant potential targets for therapeutic intervention, ultimately improving the prognosis in sHLH.

## Supplementary Material

Supplemental Material

## Data Availability

All data generated or analysed during this study have been deposited in Figshare with DOI 10.6084/m9.figshare.24572764.
