# Supplementary material for: Comprehensive evaluation of immune dysregulation in secondary hemophagocytic lymphohistiocytosis
Source: Virulence. 2024 Apr 17;15(1):2342276. doi: 10.1080/21505594.2024.2342276 (PMC11028026; doi:10.1080/21505594.2024.2342276)
Supplement: Supplemental Material [file KVIR_A_2342276_SM2524.zip › Supplementary Table1.docx]

| Supplementary Table1: The baseline results of the immune characteristics between patients with HLH and control groups. | | | | | |
| --- | --- | --- | --- | --- | --- |
| Parameters | HLH (n=77) | DC (n=20) | HC (n=36) | *P*a value | *P*b value |
| CD3+ T cells (%) | 68.320 (50.650,80.090) | 78.540 (69.840,80.180) | 67.620 (62.490,72.020) | 0.136 |  |
| CD3+ T cell counts (cells/μL) | 432.000 (213.000,745.000) | 1233.000 (701.000,1409.000) | 1458.000 (1185.000,1726.710) | <0.001 |  |
| B cells (%) | 15.020 (9.250,22.400) | 14.520 (8.080,18.910) | 10.690 (8.770,12.660) | 0.043 |  |
| B cell counts (cells/μL) | 84.000 (38.000,146.000) | 197.000 (107.000,309.000) | 286.000 (227.000,509.230) | <0.001 |  |
| CD4+ T cells (%) | 29.180 (20.860,42.110) | 42.030 (35.630,48.610) | 37.630 (31.290,44.030) | 0.003 |  |
| CD4+ T cell counts (cells/μL) | 167.000 (106.000,337.000) | 638.000 (453.000,757.000) | 778.000 (607.000,989.000) | <0.001 |  |
| CD8+ T cells (%) | 25.840 (17.890,39.540) | 22.520 (17.880,33.160) | 23.930 (17.580,27.660) | 0.213 |  |
| CD8+ T cell counts (cells/μL) | 170.000 (80.000,328.000) | 346.000 (192.000,449.000) | 544.570 (352.000,653.000) | <0.001 |  |
| Th/Ts | 1.110 (0.710,1.820) | NaN(NaN) | 1.520 (1.280,2.094) | 0.011 |  |
| NK T cells (%) | 11.190 (5.710,18.860) | 10.170 (4.720,10.880) | 21.130 (12.420,27.040) | <0.001 |  |
| NKT cell counts (cells/μL) | 83.000 (33.000,133.000) | 103.000 (59.000,151.000) | 422.000 (249.000,610.230) | <0.001 |  |
| TBNKT cell counts (cells/μL) | 646.000 (368.000,1118.000) | 1509.000 (1090.000,1769.000) | 1969.000 (1634.000,2426.000) | <0.001 |  |
| NKT cells (%) | 4.060 (2.530,5.320) | 4.850 (3.660,6.550) | 4.650 (3.240,8.510) | 0.156 |  |
| NKT cell counts (cells/μL) | 23.000 (15.000,41.000) | 70.000 (38.000,141.000) | 98.000 (68.000,175.000) | <0.001 |  |
| Th1 cells (%) | 21.800 (12.800,55.700) | 18.400 (13.600,35.200) | 24.800 (18.700,28.700) | 0.796 |  |
| Th2 cells (%) | 42.200 (27.500,63.000) | 65.600 (46.800,72.500) | 37.600 (24.700,42.500) | 0.004 |  |
| Th17 cells (%) | 11.600 (5.500,18.200) | 7.100 (3.900,9.400) | 17.400 (15.500,24.500) | <0.001 |  |
| Tfh cells (%) | 12.700 (9.800,26.400) | 20.300 (13.100,21.800) | 12.700 (10.500,15.100) | 0.185 |  |
| Tfh1 cells (%) | 26.300 (13.100,43.600) | 29.900 (23.100,35.100) | 24.500 (16.900,29.600) | 0.272 |  |
| Tfh2 cells (%) | 34.900 (23.400,43.600) | 27.900 (25.900,38.800) | 28.300 (21.800,30.400) | 0.172 |  |
| Tfh17 cells (%) | 22.200 (11.600,34.700) | 22.700 (16.200,26.300) | 36.000 (33.300,38.900) | <0.001 |  |
| Naive B cells (%) | 66.900 (48.020,77.850) | 78.600 (68.400,87.100) | 71.000 (64.620,77.800) | 0.016 |  |
| Memory B cells (%) | 10.100 (4.600,16.240) | 13.200 (6.300,18.200) | 5.940 (2.270,11.800) | 0.008 |  |
| Unswitched B cells (%) | 4.200 (2.750,7.480) | 4.200 (2.100,7.100) | 7.560 (4.260,9.290) | 0.048 |  |
| Double negative B cells (%) | 14.040 (7.150,29.130) | 3.900 (2.200,6.800) | 13.330 (8.420,21.850) | <0.001 |  |
| Plasma B cells (%) | 10.500 (2.090,32.280) | 1.200 (0.500,3.200) | 0.470 (0.290,0.990) | <0.001 |  |
| HLADR+ CD3+ T cells (%) | 19.700 (11.490,36.930) | 30.100 (19.400,38.300) | 22.200 (18.250,33.400) | 0.259 |  |
| HLADR+ CD4+ T cells (%) | 17.400 (8.870,27.510) | 17.200 (11.900,21.700) | 14.800 (11.000,18.100) | 0.531 |  |
| HLADR+ CD8+ T cells (%) | 53.660 (33.870,70.190) | 50.100 (32.200,56.300) | 42.900 (31.530,54.590) | 0.057 |  |
| CD4+ CD28+ T cells (%) | 94.700 (87.930,98.000) | 96.700 (91.000,98.500) | 95.200 (91.390,97.890) | 0.388 |  |
| CD8+ CD28+ T cells (%) | 54.420 (37.480,73.530) | 55.000 (42.100,67.100) | 51.600 (44.810,59.050) | 0.459 |  |
| Naive CD4+ T cells (%) | 31.520 (17.160,43.970) | 33.380 (17.540,41.520) | 31.050 (19.700,39.900) | 0.99 |  |
| CM CD4+ T cells (%) | 22.190 (18.050,32.690) | 29.250 (22.630,32.530) | 32.480 (24.450,37.400) | 0.005 |  |
| EM CD4+ T cells (%) | 39.100 (27.390,53.030) | 37.180 (27.390,39.060) | 34.040 (23.950,44.520) | 0.38 |  |
| EMRA CD4+ T cells (%) | 1.370 (0.490,2.980) | 1.530 (0.750,2.350) | 1.680 (1.140,2.700) | 0.421 |  |
| Naive CD8+ T cells (%) | 9.410 (3.870,22.050) | 13.990 (7.590,17.170) | 12.600 (6.080,20.400) | 0.569 |  |
| CM CD8+ T cells (%) | 1.960 (1.060,3.310) | 2.390 (1.500,3.590) | 1.310 (0.860,2.760) | 0.108 |  |
| EM CD8+ T cells (%) | 53.380 (36.730,65.130) | 49.150 (36.360,52.070) | 42.740 (30.600,51.360) | 0.044 |  |
| EMRA CD8+ T cells (%)t | 22.630 (10.450,37.990) | 36.310 (26.720,41.430) | 42.100 (33.450,53.400) | <0.001 |  |
| Treg cells (%) | 1.190 (0.610,1.960) | 1.820 (1.400,2.040) | 1.801 (1.616,2.630) | <0.001 |  |
| CD45+ RA+ Treg cells (%) | 0.140 (0.070,0.300) | 0.520 (0.310,0.860) | 0.330 (0.229,0.480) | <0.001 |  |
| CD45+ RA- Treg cells (%) | 1.000 (0.500,1.590) | 1.240 (1.020,1.400) | 1.471 (1.310,2.205) | <0.001 |  |
| NKT perforin+ cells (%) | 24.400 (12.600,35.600) | 20.700 (7.900,31.300) |  |  | 0.259 |
| NKT granzyme B+ cells (%) | 60.400 (17.600,79.100) | 28.300 (6.900,49.800) |  |  | 0.045 |
| CD8+ perforin+ cells (%) | 12.800 (5.000,19.300) | 12.000 (4.600,17.600) |  |  | 0.973 |
| CD8+ granzyme B+ cells (%) | 37.500 (18.200,73.000) | 46.600 (12.500,54.800) |  |  | 0.304 |
| NK perforin+ cells (%) | 73.800 (47.600,87.400) | 52.800 (32.500,71.300) |  |  | 0.049 |
| NK granzyme B+ cells (%) | 61.200 (21.000,91.000) | 20.200 (5.300,32.300) |  |  | <0.001 |
| Classic monocyte cells (%) | 86.200 (76.630,93.800) | 75.600 (69.800,82.600) |  |  | 0.017 |
| Intermediate monocyte cells (%) | 6.190 (2.600,12.400) | 18.300 (12.500,26.500) |  |  | <0.001 |
| Nonclassic monocyte cells (%) | 2.000 (0.800,4.100) | 7.200 (4.600,9.200) |  |  | 0.001 |
| CD4dim CD8+ T cells (%) | 0.500 (0.360,0.930) | 0.750 (0.430,1.130) |  |  | 0.279 |
| Data are presented as number (%) or median (25th - 75th percentile); HLH,hemophagocytic lymphohistiocytosis; DC, disease control; HC, healthy control; Pa, the significance among the three groups of HLH, DC and HC; Pb, the significance between HLH and DC. | | | | | |
