# Supplementary figures and images for: Comprehensive evaluation of immune dysregulation in secondary hemophagocytic lymphohistiocytosis
Source: Virulence. 2024 Apr 17;15(1):2342276. doi: 10.1080/21505594.2024.2342276 (PMC11028026; doi:10.1080/21505594.2024.2342276)

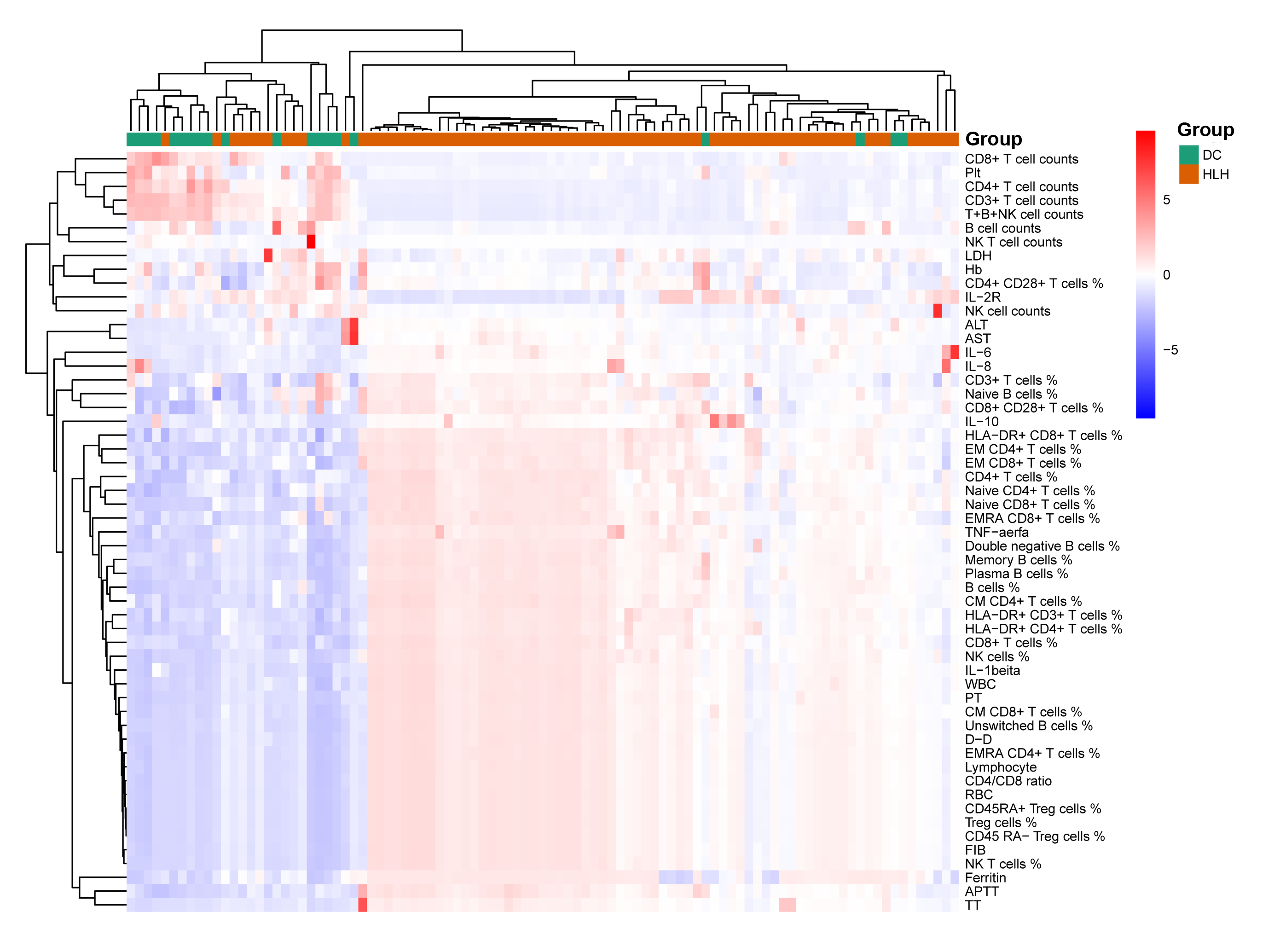

Supplement: Supplemental Material [file KVIR_A_2342276_SM2524.zip › Supplementary Figure1.tif]
